# Establishment of a prognostic risk prediction model incorporating disulfidptosis-related lncRNA for patients with prostate cancer

**DOI:** 10.1186/s12885-023-11778-2

**Published:** 2024-01-08

**Authors:** Yelisudan Mulati, Cong Lai, Jiawen Luo, Jintao Hu, Xiaoting Xu, Degeng Kong, Yunfei Xiao, Cheng Liu, Kewei Xu

**Affiliations:** 1 Department of Urology, Sun Yat-sen Memorial Hospital, Sun Yat-sen University, No. 107 Yanjiang West Road, 510000 Guangzhou, Guangdong China; 2 Guangdong Provincial Key Laboratory of Malignant Tumor Epigenetics and Gene Regulation, Sun Yat-sen Memorial Hospital, Sun Yat-sen University, 510000 Guangzhou, Guangdong China; 3 Guangdong Provincial Clinical Research Center for Urological Diseases, 510000 Guangzhou, Guangdong China; 4 Sun Yat-sen University School of Medicine, Sun Yat-sen University, 518000 Shenzhen, Guangdong China

**Keywords:** Prostate cancer, Disulfidptosis, lncRNA, Biochemical recurrence-free survival, AC026401.3

## Abstract

**Purpose:**

Prostate cancer (PCa) is one of the major tumor diseases that threaten men’s health globally, and biochemical recurrence significantly impacts its prognosis. Disulfidptosis, a recently discovered cell death mechanism triggered by intracellular disulfide accumulation leading to membrane rupture, is a new area of research in the context of PCa. Currently, its impact on PCa remains largely unexplored. This study aims to investigate the correlation between long non-coding RNAs (lncRNAs) associated with disulfidptosis and the prognosis of PCa, seeking potential connections between the two.

**Methods:**

Transcriptomic data for a PCa cohort were obtained from the Cancer Genome Atlas database. Disulfidptosis-related lncRNAs (DDRLs) were identified through differential expression and Pearson correlation analysis. DDRLs associated with biochemical recurrence-free survival (BRFS) were precisely identified using univariate Cox and LASSO regression, resulting in the development of a risk score model. Clinical factors linked to BRFS were determined through both univariate and multivariate Cox analyses. A prognostic nomogram combined the risk score with key clinical variables. Model performance was assessed using Receiver Operating Characteristic (ROC) curves, Decision Curve Analysis (DCA), and calibration curves. The functional impact of a critical DDRL was substantiated through assays involving CCK8, invasion, migration, and cell cloning. Additionally, immunohistochemical (IHC) staining for the disulfidptosis-related protein SLC7A11 was conducted.

**Results:**

The prognostic signature included AC026401.3, SNHG4, SNHG25, and U73166.1 as key components. The derived risk score from these signatures stood as one of the independent prognostic factor for PCa patients, correlating with poorer BRFS in the high-risk group. By combining the risk score with clinical variables, a practical nomogram was created, accurately predicting BRFS of PCa patients. Notably, silencing AC026401.3 significantly hindered PCa cell proliferation, invasion, migration, and colony formation. IHC staining revealed elevated expression of the dithiosulfatide-related protein SLC7A11 in tumor tissue.

**Conclusions:**

A novel prognostic signature for PCa DDRLs, possessing commendable predictive power, has been constructed, simultaneously providing potential therapeutic targets associated with disulfidptosis, among which AC026401.3 has been validated in vitro and demonstrated inhibition of PCa tumorigenesis after its silencing.

**Supplementary Information:**

The online version contains supplementary material available at 10.1186/s12885-023-11778-2.

## Introduction

PCa is the second most prevalent malignancy in men globally and the leading cause of cancer-related mortality among them [1]. Although PCa generally progresses more slowly than other malignancies, various therapeutic modalities, including surgery, chemotherapy, and hormone therapy, have enabled personalized diagnostic and treatment approaches for patients at different clinical stages and risk profiles. Nevertheless, biochemical recurrence remains a significant risk factor for the prognosis and survival of PCa patients [2] diminishing the quality of life and disease-free survival, subjecting patients to profound physical and psychological distress, as well as a substantial economic burden. Therefore, the need for accurate prognosis prediction becomes evident.

Disulfidptosis pertains to cellular demise triggered by the aberrant buildup of disulfide compounds within cells [3]. The buildup of intracellular disulfides, such as cysteine, can induce disulfide stress, leading to a high level of toxicity and harm to cells [4, 5].

LncRNAs are non-coding transcripts longer than200 bp. Although they were previously considered junk DNA, researches have increasingly recognized their key role in cell mechanisms, cell cycle regulation, differentiation and apoptosis [6, 7]. It has been reported that lncRNAs participate in regulating multiple genes and pathways in breast cancer tumorgenesis and endocrine resistance [8, 9], are related to m6A modifcation in bladder cancer affectingnearly all aspects ofRNA metabolism and arecorrelated with drug resistance in hepatocellular carcinoma via certain pathways and more [10–13].

In recent times, a multitude of investigations across diverse tumor domains have focused on developing prognostic survival models and drug resistance prediction models. These models hinge upon the disparate expression of genes or lncRNAs between tumor and its neighboring normal tissues. The outcomes have underscored the robust predictive efficacy inherent in these models. Furthermore, the incorporation of supplementary pertinent predictive determinants has exhibited the potential to heighten the precision of prognostic estimates. Liu et al. established a prognostic and chemotherapy drug sensitivity prediction model for cervical cancer based on disulfidptosis-associated lncRNAs and demonstrated excellent predictive performance [14]. Jiang et al. similarly constructed a prognostic prediction model for bladder cancer, which was based on disulfidptosis-associated lncRNAs, and they found three genes (NDUFA11, RPN1, SLC3A2) that are disulfidptosis related, which could be potential novel biomarkers for bladder cancer diagnosis and treatment [15]. Taking a more advanced approach, Wang et al. employed machine learning techniques to achieve a predictive model for breast cancer prognosis, by using genes that are disulfidptosis associated and revealed a strong connection between these genes and factors like the tumor microenvironment, infiltration of immune cells into tumors, tumor mutation burden, tumor stemness, and drug responsiveness [16].

The emergence of disulfidptosis as a recently unveiled cellular demise mechanism, along with its prospective associated factors, holds the potential to influence tumor-related behaviors. This introduces novel dimensions to forthcoming anti-tumor therapeutic strategies. Here we constructed a risk scoring formula for PCa patients, which is based on DDRLs, and simultaneously incorporated relevant clinical factors to establish a prognostic prediction nomogram model. Additionally, we conducted cellular functional validation of the crucial DDRL that indicated it being a potential target for inhibiting PCa.

## Methods

### Acquisition of disulfidptosis-related genes and lncRNAs

The TCGA database procured PCa transcriptome sequencing data and corresponding clinical information, including patient age, clinical stage, and treatment history. The dataset underwent standardization using the “edgeR” package within R software (version 3.6.3) with specific parameters set for differential expression: FDR < 0.05 and logFC ≥ 0.5. A volcano plot was constructed to depict the lncRNAs expression differences of tumor and its neighboring tissues. The disulfidptosis related genes (DDRGs) were extracted from a former study by Liu et al. [3]. The correlation between differential expressed lncRNAs and DDRGs was computed by Pearson correlation analysis with a significance threshold of *p* < 0.05. LncRNAs exhibiting a correlation coefficient |R2| > 0.3 and *p* < 0.05 were identified as having an association with disulfidptosis and subsequently chosen for further comprehensive analysis. The research flowchart for this study is provided in Supplementary material 1.

### Screening of DDRLs and predictive signature establishment

Utilizing the aforementioned screened DDRLs, we employed univariate Cox and LASSO regression analysis to ascertain DDRLs associated with BRFS, detailing the selection of lambda.min in LASSO and hazard ratio thresholds in Cox analysis. Within the framework of LASSO, the risk coefficient (coefi) was calculated associating with each individual DDRL. The risk score was calculated as the sum of the product of each DDRL’s coefficient and its expression level, with the formula: risk score=${\sum }_{i=1}^{n}(Coe{f}_{i}\times Exp{r}_{i})$ [17].

### Verification of the risk-scoring model and prognostic analysis

We obtained clinical information from 384 patients with PCa, excluding survival time less than 30 days. The samples were segregated into training and validation sets at a ratio of 2:1. With the median value as the cut-off point, patients were grouped into high- and low-risk group. Survival analysis was executed with the ‘survival’ R-package to ascertain the presence of a significant discrepancy in BRFS between high-risk and low-risk patients. Time dependent ROC curves were generated over time using the ‘timeROC’ package. The risk scoring model’s predictive performance was evaluated by the area under the curve (AUC). Univariate and multivariate Cox regression analyses were conducted to elucidate the interrelationships among risk scores, age, TNM staging, Gleason score, and BRFS in PCa patients.

### Construction and effectiveness evaluation of nomogram prediction model

The ‘rms’ package was utilized to construct a nomogram that amalgamated age, T and N stage, Gleason and risk score, facilitating the prediction of 1-, 3-, and 5-year BRFS in PCa patients. Subsequently, we conducted separate assessments of the efficacy of this nomogram prediction model on both training and validation sets. The 1-, 3- and 5-year nomogram calibration curve were used to evaluate the accuracy of the nomogram predictive model. DCA was conducted to evaluate net benifit of the nomogram predicted 1-, 3- and 5-year BRFS. Evaluation of the nomogram model’s predictive ability was conducted through ROC curve analysis.

### Cell lines and cell culture

Human PCa cell lines DU145 and PC3 were kindly provided by Cell Bank, Chinese Academy of Sciences and cultivated in RPM-1640 and DMEM media (Gibco, NY, USA). The growth medium was supplemented with 10% fetal bovine serum (FBS, BI, Israel), together with 100 U/ml penicillin and 100 mg/ml streptomycin. Incubation was carried out in a humidified CO_2_ incubator at 37 °C.

### RNA interference

GenePharma (Suzhou, China) synthesized and purified the specific small interfering RNA (siRNA) duplexes. DU145 and PC3 cells were seeded into six-well plates at a density of 1 × 10^5^ cells and were divided into the control group and the siRNA group. They were cultured until they reached a confluence of 50-70%. Transient transfection of the siRNA and negative control was performed using Lipofectamine iMAX (Invitrogen, USA). The sequences of siRNA and its negative control can be found in Supplementary Material 2.

### cDNA synthesis and qRT-PCR

The PrimeScript RT Reagent Kit (Takara, Shiga, Japan) synthesized complementary DNA (cDNA) for gene expression analysis Subsequently, qRT-PCR was conducted by combining the cDNA samples with TB Green Premix Ex Taq II (Takara, Shiga, Japan). the 2^-ΔΔCT method helped determine the relative expression levels. GAPDH served as an internal control for mRNA expression. The sequences of primers for AC026401.3 and GAPDH can be found in Supplementary Material 2.

### CCK-8 assay

The CCK-8 assay was used to assess the cell viability of DU145 and PC3 cell lines in the control group and siRNA group following transfection. Cells were seeded with a density of 2000 cells per well in 96-well plates with 100 µL of medium followed by siRNA transfection after 48 h. The CCK-8 assay was conducted 24 h after transfection with 10ul of CCK-8 solution mixed with 90ul medium introdued to each well. Subsequent to an incubation of 2 h, the optical density (OD) at 450 nm was measured by a Tecan Spark 10 M multimode microplate reader (Tecan, Austria) to assess cell proliferation and viability. This process was done in triplicate and repeated three times. Cell viability (%) was computed using the formula: (cell number difference between the experimental and blank group) / (cell number difference between the control and blank group)×100.

### Transwell assay

A suspension of 50,000 cells was introduced in the upper chamber, with 200 µL of serum-free medium to facilitate migration, supplemented with 200 µL of matrigel to promote invasion. The medium containing 10% FBS was introduced into the lower chamber, totaling 600 µL.48 h later, samples obtained from the upper chamber were harvested, followed by fixation in 4% paraformaldehyde for 20 min, and by subsequent staining using 0.1% crystal violet. Cell quantification was used for the cells that had penetrated, and imaged by a Nikon Ni-U microscope (Nikon, Japan). 200 cells were seeded into each well of six-well plate, and cultured for 2 weeks, then the colonies were analyzed.

### SLC7A11 IHC staining

Paraffin sections from PCa and adjacent tissues were made available through the generosity of Sun Yat-sen Memorial Hospital of Sun Yat-sen University. Immunohistochemistry (IHC) staining on rehydrated sections was done according to established methods [18]. Photomicrographs were captured using a Nikon Ni-U microscope (Nikon, Japan). The antibodies used in the study, along with their respective dilutions, were obtained from SAB and Abcam: anti-SLC7A11 antibody (43,437, 1:1000) and goat anti-rabbit IgG H&L (HRP) (ab6721, 1:500).

### Statistical analysis

Statistical analyses were performed through R software and GraphPad Prism (version 7.03).

## Results

### Primary screening and identification of DDRLs with differential expression

**Fig. 1:**
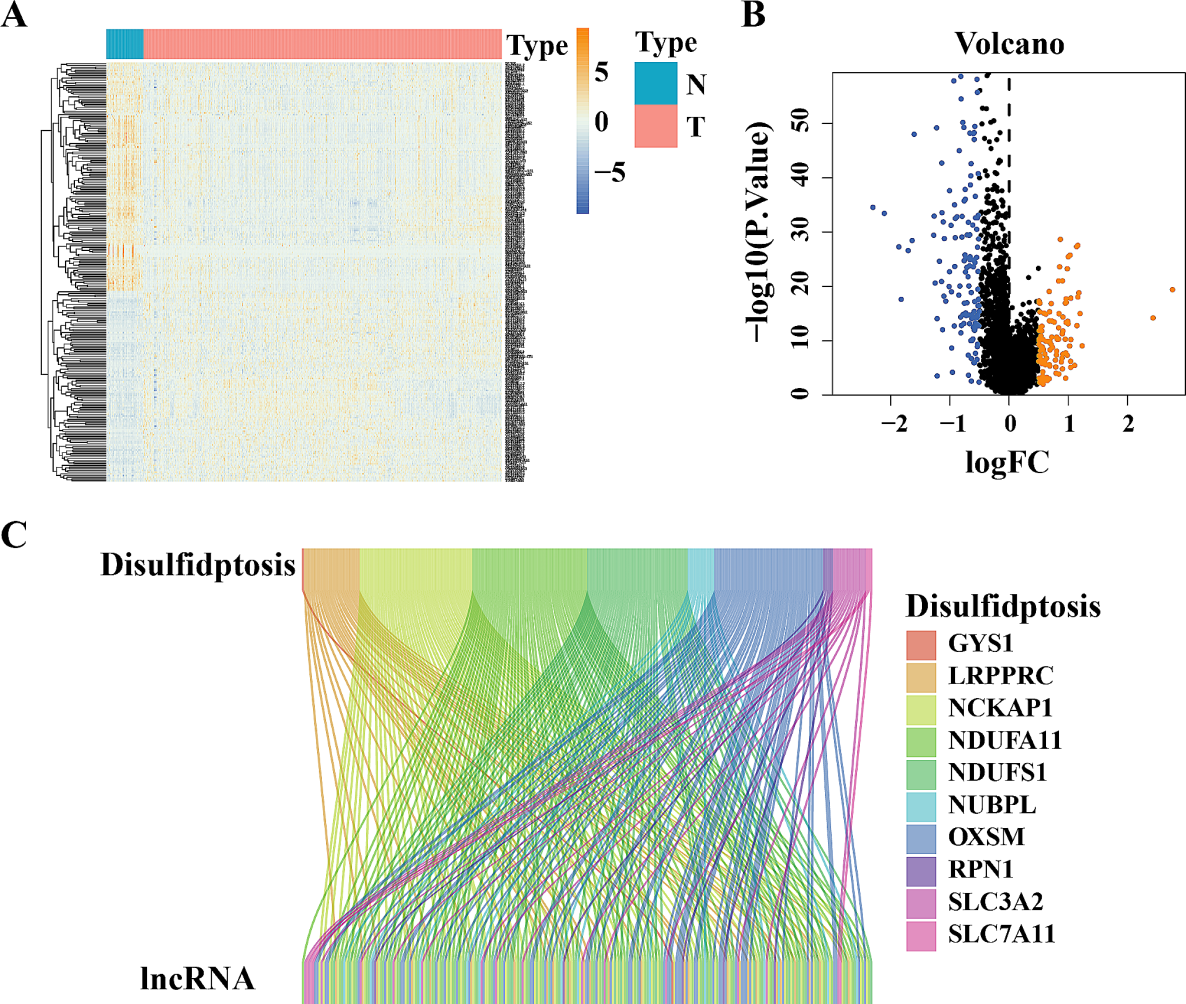
Identification of disulfidptosis-related lncRNAs in prostate cancer cohort. (**A**) Heatmap of the differentially expressed lncRNAs showing expression signature in patients with PCa. (**B**) Volcano plot of differentially expressed lncRNAs. (**C**) Sankey diagram of disulfidptosis-related lncRNAs.

Transcriptome sequencing data along with comprehensive clinical follow-up details were acquired from 384 patients diagnosed with PCa, sourced from the TCGA database. Differentially expressed lncRNA profile was generated by the ‘limma’ R package (Fig. 1A, B). 10 DDRGs were obtained from previously reported literature [3]. A correlation analysis was conducted between lncRNA profile and 10 DDRGs and 112 DDRLs were identified (Fig. 1C).

### Identifying candidate DDRLs associated with the BRFS of PCa patients

**Fig. 2:**
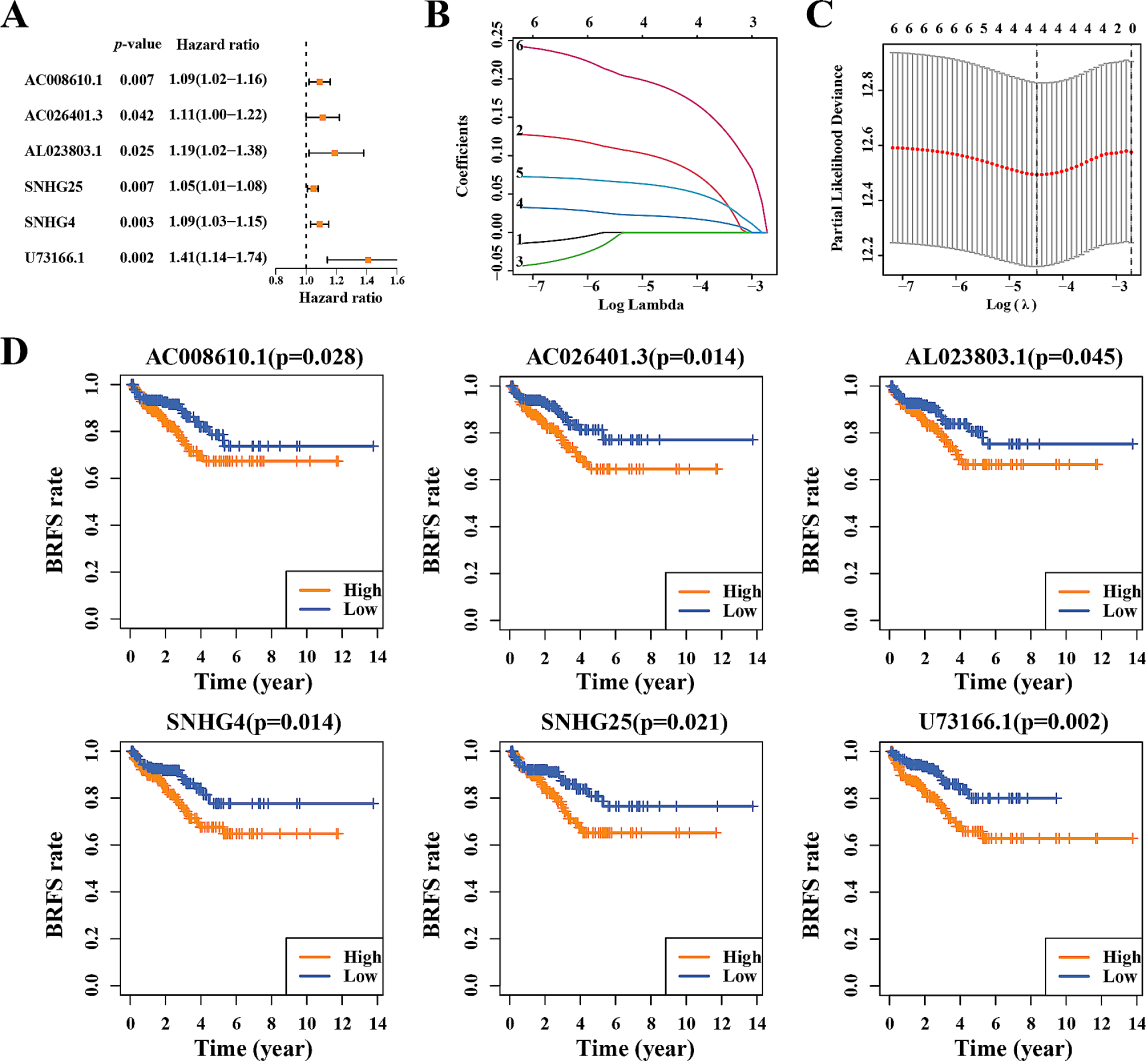
Identifying candidate disulfidptosis-related lncRNAs associated with the Biochemical Recurrence-Free Survival of prostate cancer patients. Biochemical Recurrence-Free Survival of AC008610.1, AC026401.3, AL023803.1, SNHG4, SNHG25 and U73166.1 in TCGA. (**A**) Univariate Cox regression analysis of disulfidptosis-related lncRNAs. (**B, C**) Determination of the best penalty value. LASSO regression of screening disulfidptosis-related lncRNAs with patients’ prognosis. (**D**) Biochemical Recurrence-Free Survival of AC008610.1, AC026401.3, AL023803.1, SNHG4, SNHG25 and U73166.1 in TCGA

The role of DDRLs were preliminarily examined through initial univariate Cox regression analysis, aiming at a comprehensive grasp of their significance. The results indicated that 6 DDRLs negatively associated with the BRFS of PCa Patients (Fig. 2A). BRFS analysis of these 6 DDRLs in the TCGA cohort were shown in Fig. 2D. To mitigate the potential influence of overfitting on result authenticity, LASSO regression was used to further refine the selection of the 6 DDRLs. As a result, 4 lncRNAs (AC026401.3, SNHG4, SNHG25 and U73166.1) were filtered (Fig. 2B and C). The expression levels of the 4 DDRLs in the TCGA datasets can be found in Supplementary Material 3.

### Development of a risk score model utilizing differentially expressed and BRFS-Associated DDRLs

**Fig. 3:**
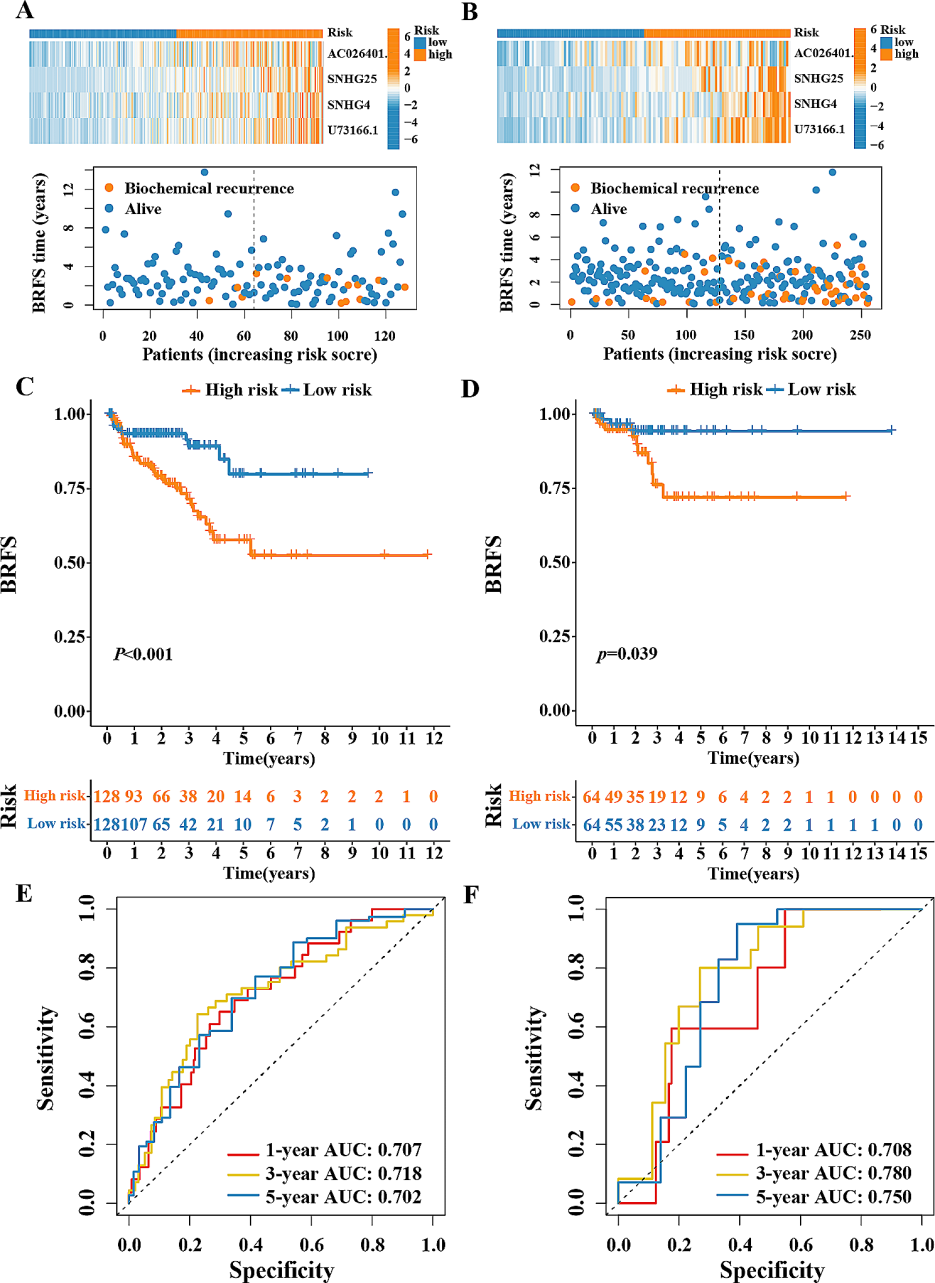
Validation of risk score through data derived from TCGA. The distribution of the four disulfidptosis-related lncRNAs risk score and survival status for each patient in training set (**A**) and validation set (**B**); Kaplan–Meier curve of survival time in high- and low-risk groups in training set (**C**) and validation set (**D**); ROC curve of 1-, 3- and 5-year BRFS in training set (**E**) and validation set (**F**)

Utilizing the given expression (Expr) and coefficient value of each DDRL (Table 1), a risk scoring model was formulated: risk score=${\sum }_{i=1}^{n}(Coe{f}_{i}\times Exp{r}_{i})$. Using the individual results, the PCa training and validation sets derived from TCGA were categorized into high-risk and low-risk groups based on the median value. The heat map visualization illustrates distinct expression patterns of the four DDRLs in both groups. (Figure 3A and B). As anticipated, the high-risk group exhibited a higher mortality rate compared to the other. (Figure 3C and D). The evidence supports the notion that the risk scoring model exhibited strong predictive capabilities, indicating a more favorable prognosis for individuals in the low-risk group compared to the high one. ROC curves showed great predicting efficacy of the model in 1-, 3-, 5-year BRFS rate predicting both on the training and the validation sets (Fig. 3E and F).

**Table 1:** The detailed information of the four disulfidptosis-related lncRNA for the prediction model

LncRNA	HR	Coef	*p*-value	
AC026401.3	1.105	0.093	0.042	
SNHG25	1.048	0.020	0.007	
SNHG4	1.090	0.059	0.003	
U73166.1	1.406	0.186	0.002	

### Prognostic predicting nomogram establishment

**Fig. 4:**
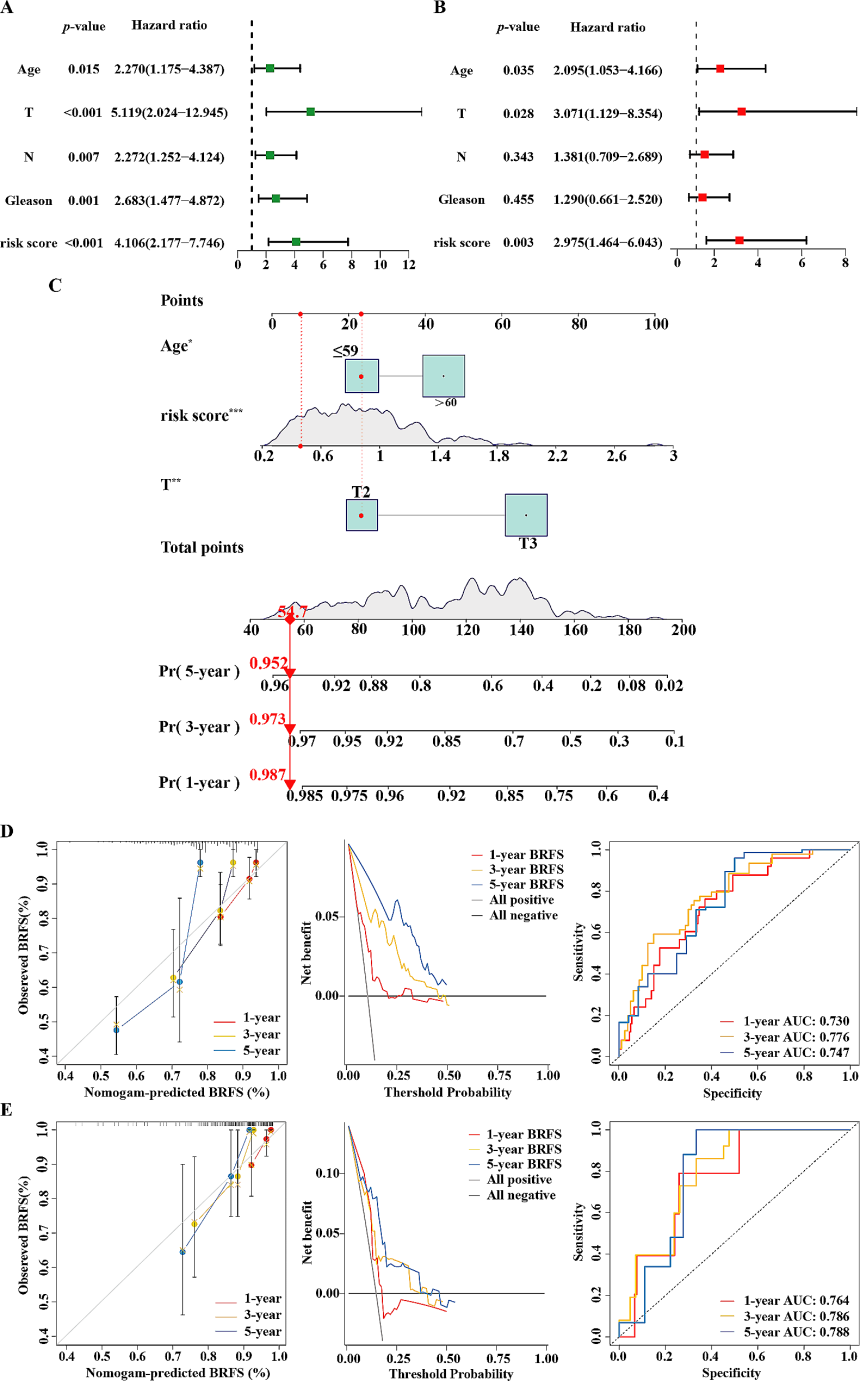
Prognostic Nomogram Establishment. Univariate (**A**) and multivariate (**B**) Cox regression analysis of clinical information (Age, T stage, N stage, Gleason score and risk score). (**C**) The prognostic nomogram that combines clinicopathological factors and risk scores predicts the 1-, 3-, and 5-year BRFS of PCa patients. Calibration curves, the Decision Curve Analysis (DCA) curves and ROC curves evaluating the prognostic efficiency of the very model in the training set (**D**) and validation set (**E**), respectively

To further investigate possible predictive clinical fators, survival analysis was performed in different clnicopathological subgroups of PCa patients. Baseline clinical data of patient populations in the training set and validation set can be found in table S1. Samples with missing information were excluded. The outcome highlighted a discernible difference in survival status between both groups in relation to age (≤ 60, >60), T stage (≤T_2_, ≥T_3_) and Gleason score (≤ 7, ≥ 8) (Supplementary material 4). Univariate Cox analysis suggests that age, T and N stage, Gleason score and risk score were risk factors for BRFS of PCa patients (Fig. 4A). Also, multivariate Cox analysis ascertained that age, T stage and risk score were independent risk factors for PCa patients’ BRFS (Fig. 4B). Thus, a prognostic nomogram was created utilizing age, T stage and risk score (Fig. 4C). Moreover, we conducted separate assessments of the efficacy of this nomogram prediction model on both the training set (Fig. 4D) and the validation set (Fig. 4E). Calibration curves for nomograms to predict the 1-, 3- and 5-year BRFS of PCa patients showed satisfactory accuracy of the nomogram predictive model. DCA cruves for nomograms to predict the 1-, 3- and 5-year BRFS of PCa patients showed that the model was of great clinical benifit for PCa patients. ROC curve verified good efficacy of the predicting model.

### AC026401.3 knockdown inhibited proliferation of PCa cells

**Fig. 5:**
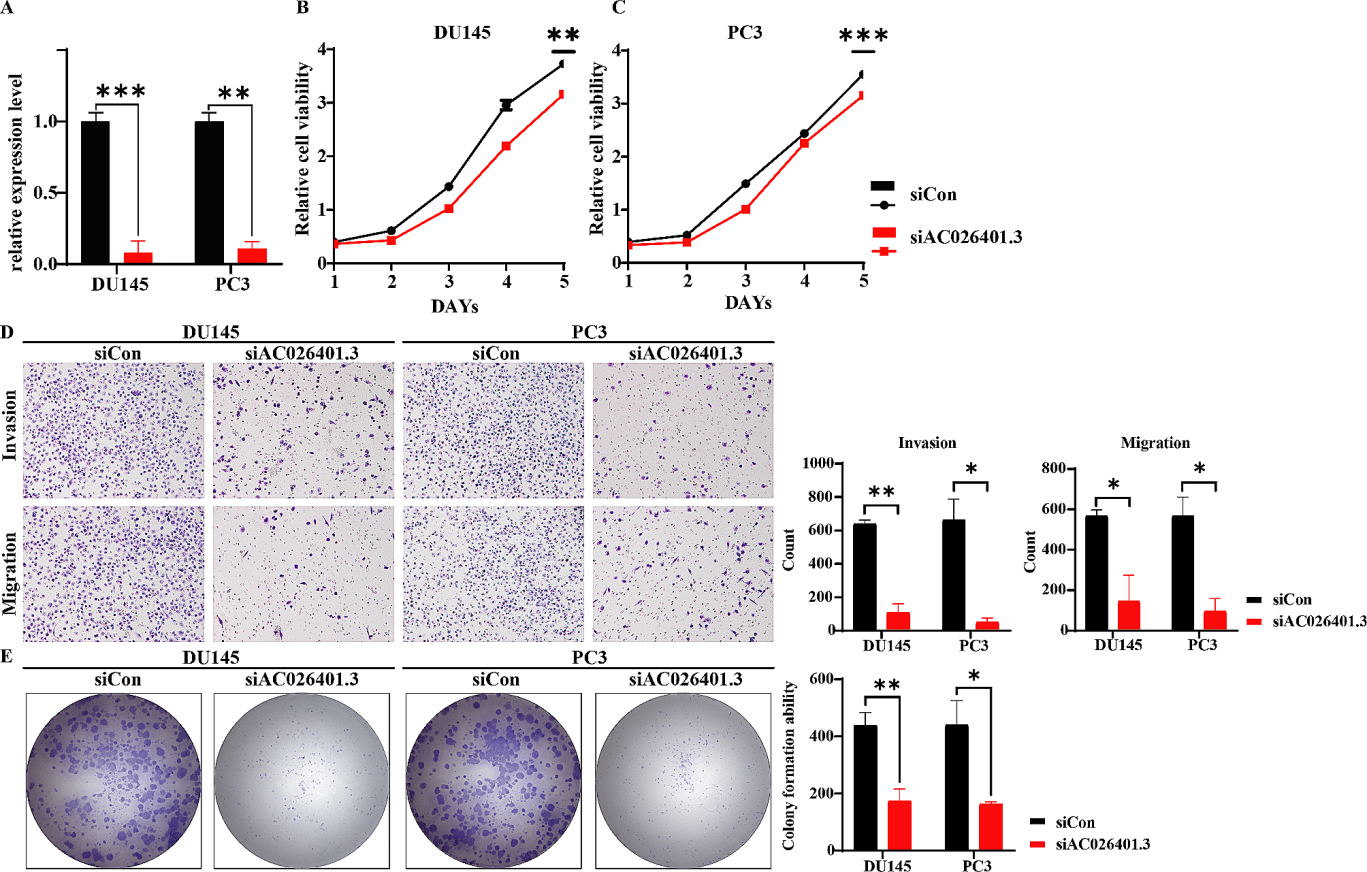
Cellular Functional Validation of AC026401.3. (**A**) Transfection efficiency of the control group and the siRNA group in both cells. (**B, C**) CCK-8 assay results indicate lower cell viability in the siRNA group compared to the control group in both cells. The *p*-value for the fifth day is noted. (**D**) Significantly reduced invasion and migration capabilities were observed in the siRNA group compared to the control group through invasion and migration assays in both cells. (**E**) Significantly decreasing of colony-forming ability was observed in the siRNA group compared to the control group in the cell cloning experiments. (*, *p* < 0.05; **, *p* < 0.01; ***, *p* < 0.001)

To investigate the impact of AC026401.3 on cellular functions, we reduced its expression in PC3 and DU145 cancer cells. We confirmed a significant decrease in the expression level of AC026401.3 in the siAC026401.3 group compared to the siCon group using RT-qPCR (Fig. 5A). CCK-8 assay results demonstrated lower cell viability in DU145 and PC3 cells of the siAC026401.3 group compared to the siCon group (Fig. 5B, C). Furthermore, the migratory, invasive, and colony-forming abilities of cells in the siAC026401.3 group were significantly reduced (Fig. 5D, E).

### Variance in SLC7A11 expression between PCa tissue and benign prostate tissue adjacent to tumor

**Fig. 6:**
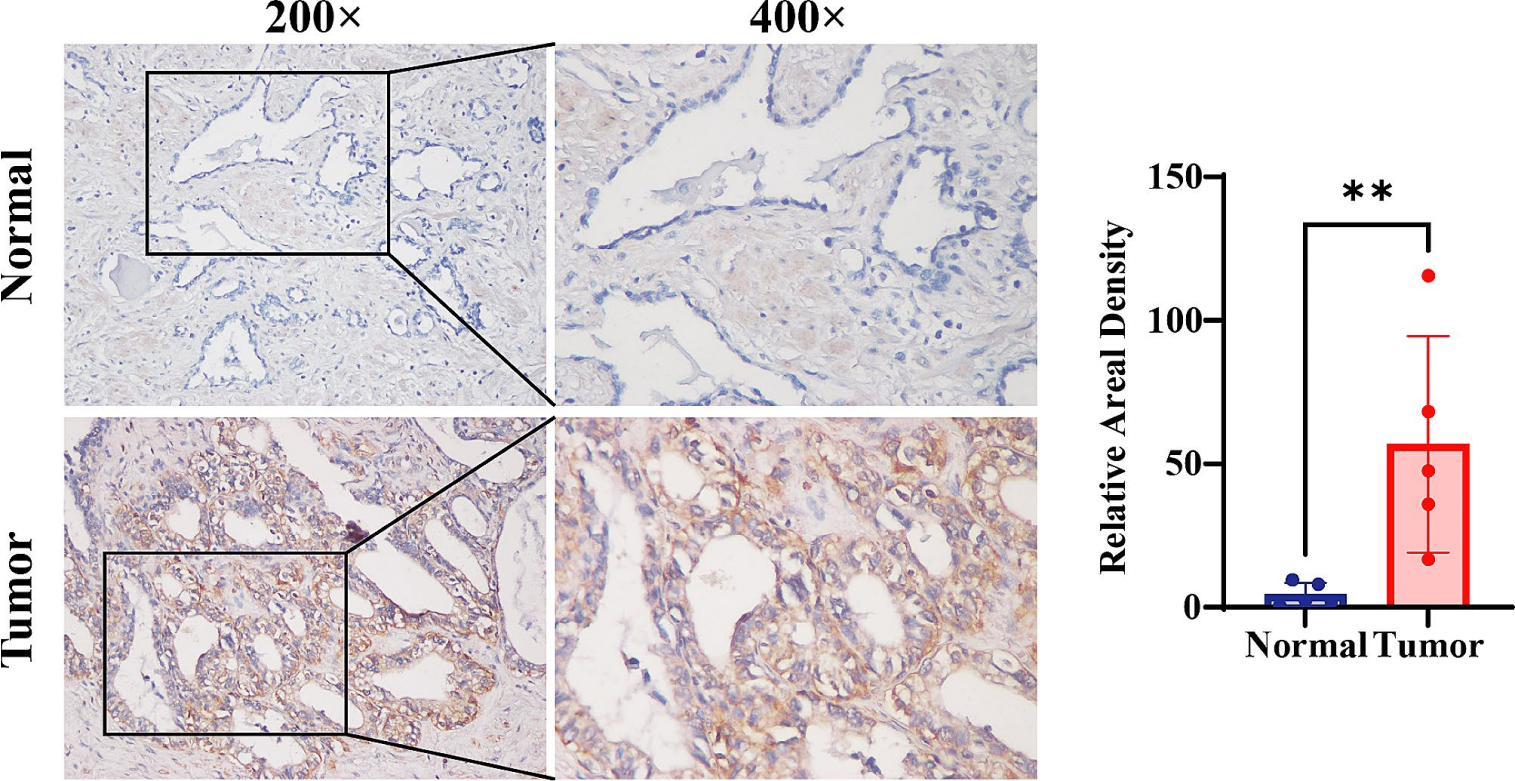
Immunohistochemical analysis of SLC7A11 expression in prostate tissues. Representative micrographs of normal (upper panels) and tumor (lower panels) prostate tissues stained for SLC7A11 at 200x and 400x magnification (left and right in each tissue type, respectively). Insets show higher magnification of the selected areas. The bar graph on the right quantifies the relative areal density of SLC7A11 staining, with significant increase in tumor tissue compared to normal (**, *p* < 0.01)

Moreover, we performed SLC7A11 IHC staining to detect the expression of SLC7A11 in four pairs of matched pathological section of PCa and benign prostate tissue adjacent to tumor from PCa patients. As shown in Fig. 6, in cancer tissues, the expression of SLC7A11 tended to be higher.

## Discussion

As high-throughput sequencing technologies are progressively adopted, the increasingly abundant genomic sequencing data not only offers novel potential targets for tumor therapy, but also introduces new foundations for tumor diagnosis and prognostic assessment through the differentially expressed genes identification.

Although currently, a comprehensive range of assessment strategies, including clinical staging, Gleason score, PSA risk stratification, and pathological staging, provide necessary foundations for individualized treatment decisions in PCa for most patients, the prognosis of treatment remains a significant challenge. For instance, the effective monitoring of minuscule recurrent and metastatic lesions in PCa, coupled with the challenges posed by biochemical relapse, presents a formidable hurdle, thereby tempering the optimism surrounding treatment prognosis [19]. There are also reports that, despite undergoing active treatment, approximately 50% patients of high-risk PCa still experience biochemical recurrence [20]. This could be attributed to significant individual variations in PCa, implying that not all patients can equally benefit from the same treatment approach [21]. Therefore, conducting individual prognostic prediction for PCa patients appears to hold certain clinical significance.

Liu et al. in their research, originally reported a distinct cell death form that differs from apoptosis and ferroptosis, which termed disulfidptosis. Their findings regarding the sensitization of cell membranes to disulfidptosis indicate that intracellular disulfide accumulation pressure can induce stress in actin cytoskeleton proteins of SLC7A11-overexpressing renal cancer cells, resulting in the formation of abnormal disulfide bonds among actin cytoskeleton proteins. This phenomenon ensues from the anomalous expression of the cystine transporter, namely solute carrier family 7 member 11 (SLC7A11), colloquially identified as xCT. Heightened rates of cystine uptake and subsequent enzymatic reduction to cysteine, in conjunction with concurrent glucose deprivation, precipitate an exhaustive consumption of the intracellular nicotinamide adenine dinucleotide phosphate (NADPH) reservoir. This depletion eventuates in the prolific intracellular accrual of disulfide moieties, thereby eliciting swift apoptotic demise of the affected cells [3]. Insufficient NADPH supply caused by glucose deprivation, which also contributed to the cell disulfidptosis endin, leading to the loss of the reducing power required to resolve these abnormal disulfide bonds. It is worth noting that under normal conditions, the glucose pentose phosphate pathway produces NAPDH, which can resist the toxicity of disulfide accumulation, convert it non-toxic, and maintain the cellular inventory. Without a doubt, their research findings have suggested a novel cell death mechanism with the potential to become a new avenue for anti-tumor therapy. Based on their whole-genome CRISPR-Cas9 screening, demonstrated 10 hits associated with disulfidptosis. Among them, SLC7A11, which can transport cystine from outside the cell into the cell, providing the cell with the raw material for the synthesis of glutathione for antioxidant defense [22, 23], has been demonstrated in another study to exhibit differential expression in various tumors. Its expression level dictates the sensitivity of tumor cells to oxidative stress [24]; Zhang et al. in their study, utilized clear cell renal cell carcinoma (ccRCC) cohort data to screen and filter out four disulfidptosis-associated metabolic genes that are correlated with prognosis. Concurrently, an inquiry was undertaken to delve into the conceivable mechanistic implications of SLC7A11, alongside its pertinent implications for patient prognostication within the cohort under investigation. The ensuing findings unequivocally underscore that escalated expression levels of SLC7A11 correlate conspicuously with compromised overall survival (OS), a abbreviated progression-free interval (PFI), and constrained disease-specific survival (DSS) within the subset afflicted by ccRCC. Furthermore, a marked upregulation of SLC7A11 was conspicuous among subjects who experienced mortality, as well as within the neoplastic tissue specimens. These cumulative observations irrefutably validate the pronounced clinical significance of SLC7A11, affirming its utility as an astute prognostic biomarker specific to ccRCC and conclusively anchoring its interrelation with the course of disease progression [25]. In addition to SLC7A11, in Li et al.‘s validation results from whole-genome CRISPR-Cas9 screening, SLC3A2 (encoding a partner of SLC7A11), RPN1, and NCKAP1 were identified as other inhibitory factors. Knockdown of RPN1 conferred increased resistance of UMRC6 renal cancer cells to disulfidptosis, whereas NCKAP1 slowed down the formation of disulfide bonds induced by glucose starvation and facilitated the detachment of f-actin contraction and depolymerization from the cell membrane in UMRC6 cells, thus providing some degree of protection to the cellular cytoskeleton. In order to further validate the differential expression of disulfidptosis-associated proteins between PCa and adjacent normal prostate tissues, we conducted immunohistochemical validation of SLC7A11 and obtained results consistent with previous research findings.

In the prognosis prediction of PCa, age, Gleason score, and TNM staging each hold significant clinical importance. Older patients typically have a poorer prognosis, possibly due to more comorbidities, less frequent detection through PSA testing, more advanced cancer stages, and less frequent curative treatments [26]. The Gleason score assesses the histological grade of the tumor and is a crucial indicator of the malignancy of PCa [27]. The TNM staging system classifies cancer based on tumor size and spread (T), lymph node involvement (N), and the presence of metastasis (M), with its revisions emphasizing a shift from anatomical to biological classification [28]. Together, these three factors provide a critical basis for treatment choices and management strategies in PCa. Our risk scoring model was constructed based on four DDRL exhibitting a strong correlation with BRFS of PCa. In constructing the BRFS prediction nomogram, the results of clinical subgroup analysis were also incorporated, including certain clinical indicators, which significantly enhanced the predictive efficacy of the model. Subsequent validation of this risk scoring model revealed not only a robust capacity for evaluation but also demonstrated favorable performance. In previous studies, AC026401.3 have been demonstrated to be associated with various oncological behaviors, including tumor lymphocyte infiltration, cuproptosis, senescence, ferroptosis and so on [29–31]. Among which a noteworthy study on this matter comes from Cao et al. In their research, they built a risk score by a formula based on 6 significantly related glycolysis-based lnc RNAs where AC026401.3 was among with. And their risk score signature among with their prognostic predictive nomogram for patients with renal cell carcinoma were evaluated of good performance. The employment of Gene Set Enrichment Analysis (GSEA) in this study also highlight the potential involvement of the glycolysis-related lncRNA signature in modulating immune-related processes [32]. Unfortunately, they did not conduct relevant functional validation. Based on these research findings, in this study, we conducted in vitro functional validation of AC026401.3, identified as a key DDRL. The results demonstrated that knockdown of AC026401.3 could inhibit cell viability, migration, and invasion capabilities of both DU145 and PC3 PCa cell lines. Additionally, it effectively suppressed the formation of clonal colonies in these two PCa cell lines. Hence, AC026401.3 holds potential as a target for future anti-cancer therapy in PCa. What’s even more exciting is that, in connection with our SLC7A11 IHC staining, we speculate that the observed antitumor effect due to AC026401.3 knockdown could be mediated through its impact on intracellular glycolysis. As mentioned earlier, cancer cells with high SLC7A11 expression face pressure from intracellular disulfide accumulation under glucose starvation, leading to disulfidptosis and AC026401.3 was also among the glycolysis-based lnc RNAs as Cao et al. identified before. Could the correlation of AC026401.3 with disulfidptosis be triggered by its glycolysis-related effects on cancer cells? This aspect warrants further exploration in future research endeavors.

## Conclusion

Through the examination of TCGA cohorts’ transcriptome signature, a risk scoring system was developed based on four DDRLs, which demonstrated exceptional ability in predicting the prognosis of individual patients with PCa. Furthermore, functional assays conducted in vitro suggested that AC026401.3, an essential DDRL, exhibited inhibitory effects on cell viability, invasion, and migration in PCa cell lines. Meanwhile, IHC verified the differential expression of SLC7A11 in PCa tissues and adjacent normal tissues, which indicated the potential of inhibitting tumor growth by triggering disulfidptosis. Our results suggest that the risk score model may have implications as a prognostic marker for individuals with PCa, and the BRFS prediction nomogram could be a valuable tool for evaluating post-therapy response. In the future, with more extensive external validation and in-depth functional verification, and even through further exploration of the involvement of DDRLs in the disulfidptosis mechanism, new insights may be provided for addressing a range of challenges in PCa, such as heterogeneity and drug resistance.

### Electronic supplementary material

Below is the link to the electronic supplementary material.

Supplementary Material 1

Supplementary Material 2

Supplementary Material 3

Supplementary Material 4

Supplementary Material 5

## Data Availability

Publicly available datasets were analyzed in this study: The Cancer Genome Atlas (https://portal.gdc.cancer.gov/). The datasets used and/or analysed during the current study available from the corresponding author on reasonable request.
