# Supplementary material for: Establishment of a prognostic risk prediction model incorporating disulfidptosis-related lncRNA for patients with prostate cancer
Source: BMC Cancer. 2024 Jan 8;24:44. doi: 10.1186/s12885-023-11778-2 (PMC10775669; doi:10.1186/s12885-023-11778-2)
Supplement: Supplementary file 1 — Supplementary Material 1 [file 12885_2023_11778_MOESM1_ESM.docx]

**Supplementary material 2. The sequences of siRNA and its negative control, and primers for AC026401.3 and GAPDH.**

si-AC026401.3

sense（5'-3'）:

UCAAUGAGAAGCUAAGAAGCA

antisense（5'-3'）

CUUCUUAGCUUCUCAUUGAGA

Negative Control (NC)

sense（5'-3'）:

UUCUCCGAACGUGUCACGUTT

antisense（5'-3'）

ACGUGACACGUUCGGAGAATT

Primer：

| LncRNA AC026401.3 | F-CCCACAGAGTGGTGAAAAACTT |
| --- | --- |
|  | R-ACAAACACATCCCGGATACATT |
|  |  |
| GAPDH | F-ACCCCTTCATTGACCTCAACT |
|  | R-TGAGTCCTTCCACGATACCAA |
