# Supplementary figures and images for: Establishment of a prognostic risk prediction model incorporating disulfidptosis-related lncRNA for patients with prostate cancer
Source: BMC Cancer. 2024 Jan 8;24:44. doi: 10.1186/s12885-023-11778-2 (PMC10775669; doi:10.1186/s12885-023-11778-2)

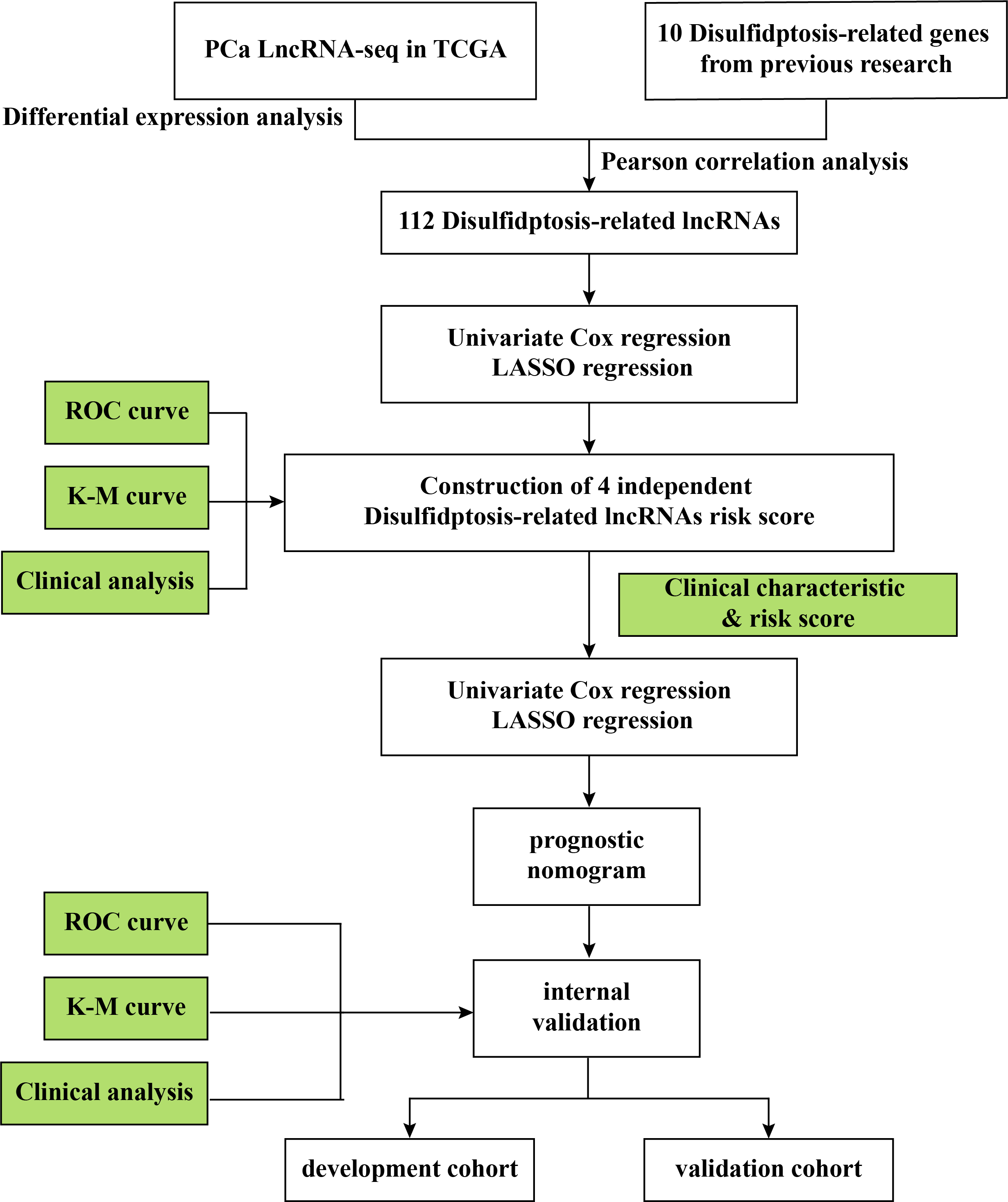


**Supplementary material 1. The flowchart of the study.**

Supplement: Supplementary file 3 — Supplementary Material 3 [file 12885_2023_11778_MOESM3_ESM.doc]
