# Supplementary material for: Establishment of a prognostic risk prediction model incorporating disulfidptosis-related lncRNA for patients with prostate cancer
Source: BMC Cancer. 2024 Jan 8;24:44. doi: 10.1186/s12885-023-11778-2 (PMC10775669; doi:10.1186/s12885-023-11778-2)
Supplement: Supplementary file 4 — Supplementary Material 4 [file 12885_2023_11778_MOESM4_ESM.doc]

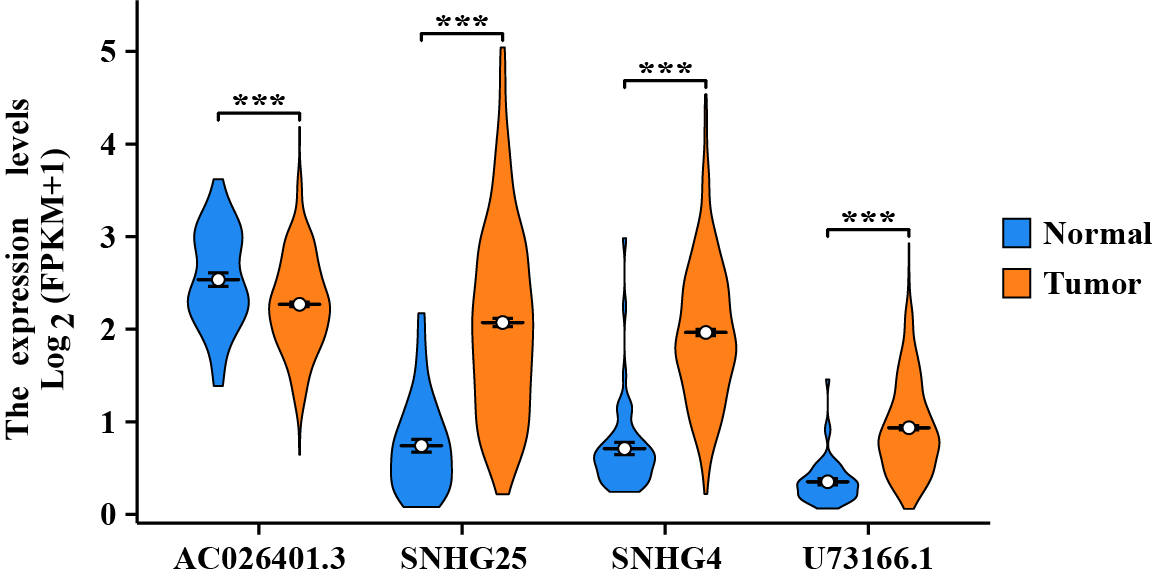


**Supplementary material 3. The expression levels of the 4 disulfidptosis-related lncRNAs in the TCGA datasets.**
