# Supplementary material for: Establishment of a prognostic risk prediction model incorporating disulfidptosis-related lncRNA for patients with prostate cancer
Source: BMC Cancer. 2024 Jan 8;24:44. doi: 10.1186/s12885-023-11778-2 (PMC10775669; doi:10.1186/s12885-023-11778-2)
Supplement: Supplementary file 5 — Supplementary Material 5 [file 12885_2023_11778_MOESM5_ESM.doc]

**
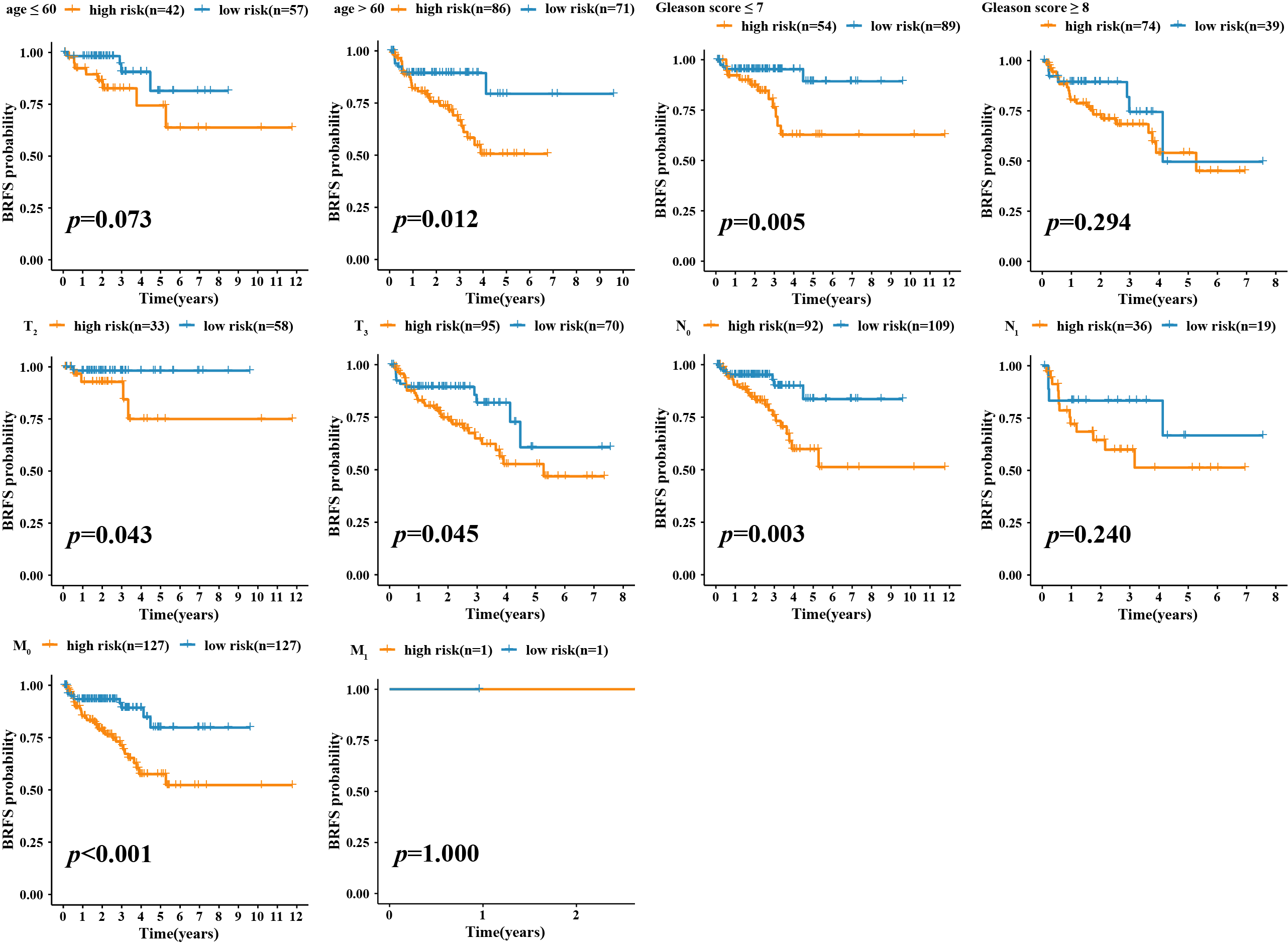
**

**Supplementary material 4. Kaplan-Meier survival analysis illustrating the prognostic efficacy of the risk score in different patient subgroups.** BRFS probability is plotted over time, comparing high-risk (indicated by crosses) and low-risk (indicated by circles) groups. Notable variance in survival outcomes is observed. In some subgroups, a significant distinction is noted (p<0.001), indicating a strong prognostic value of the risk score. In contrast, other subgroups show no significant predictive difference (p=1.000 and p=0.294). The analysis underscores the heterogeneity in risk score performance across various clinical and demographic patient subgroups.
